# Biochar: An emerging recipe for designing sustainable horticulture under climate change scenarios

**DOI:** 10.3389/fpls.2022.1018646

**Published:** 2022-12-05

**Authors:** Faisal Zulfiqar, Anam Moosa, Muhammad Mudassir Nazir, Antonio Ferrante, Muhammad Ashraf, Muhammad Nafees, Jianjun Chen, Anastasios Darras, Kadambot H.M. Siddique

**Affiliations:** ^1^ Department of Horticultural Sciences, Faculty of Agriculture and Environment, The Islamia University of Bahawalpur, Bahawalpur, Pakistan; ^2^ Department of Plant Pathology, Faculty of Agriculture and Environment, The Islamia University of Bahawalpur, Bahawalpur, Pakistan; ^3^ Department of Agronomy, College of Agriculture and Biotechnology, Zhejiang University, Hangzhou, China; ^4^ Department of Agricultural and Environmental Sciences, Università degli Studi di Milano, Milan, Italy; ^5^ Institute of Molecular Biology and Biotechnology, The University of Lahore, Lahore, Pakistan; ^6^ Mid-Florida Research and Education Center, Environmental Horticulture Department, Institute of Food and Agricultural Science, University of Florida, Apopka, FL, United States; ^7^ Department of Agriculture, University of the Peloponnese, Kalamata, Greece; ^8^ The UWA Institute of Agriculture, The University of Western Australia, Perth, WA, Australia

**Keywords:** sustainability, pyrolysis, abiotic stresses, oxidative stress, sustainable agriculture, growing media

## Abstract

The interest in sustainable horticulture has recently increased, given anthropogenic climate change. The increasing global population will exacerbate the climate change situation induced by human activities. This will elevate global food demands and the vulnerability of horticultural systems, with severe concerns related to natural resource availability and usage. Sustainable horticulture involves adopting eco-friendly strategies to boost yields while maintaining environmental conservation. Biochar (BC), a carbon-rich material, is widely used in farming to improve soil physical and chemical properties and as an organic substitute for peat in growing media. BC amendments to soil or growing media improve seedling growth, increase photosynthetic pigments, and enhances photosynthesis, thus improving crop productivity. Soil BC incorporation improves abiotic and biotic stress tolerance, which are significant constraints in horticulture. BC application also improves disease control to an acceptable level or enhance plant resistance to pathogens. Moreover, BC amendments in contaminated soil decrease the uptake of potentially hazardous metals, thus minimizing their harmful effects on humans. This review summarizes the most recent knowledge related to BC use in sustainable horticulture. This includes the effect of BC on enhancing horticultural crop production and inducing resistance to major abiotic and biotic stresses. It also discuss major gaps and future directions for exploiting BC technology.

## Introduction

Horticulture is a vital multidisciplinary sector that differs from common agriculture in terms of crops, more intensive production systems, and final profit margins. Horticultural crops include fruits and nuts, vegetables and herbs, ornamental flowering plants, trees and shrubs, lawns, and medicinal plants. Some crops are grown in containers on substrates or in growing media in greenhouses. Container substrates largely comprise organic matter, including peat moss, pine bark, coconut fiber (coir), and composted materials (Chen and Wei, 2018). Some crops are grown in soils under protected culture, and others are grown in open fields. The sector is progressively moving toward “sustainable intensification,” producing more biomass or food while maintaining environmental sustainability. Horticultural intensification is urgently needed as the global food demand between 2010 and 2050 is projected to increase by 35%–56% (van Dijk et al., 2021). Moreover, climate change is expected given the planet’s steadily growing human population. Therefore, advanced restoration approaches are required because various anthropogenic activities have increased greenhouse gas (GHG) emissions and temperatures, resulting in abnormal precipitation patterns and other climate disturbances (Sharma et al., 2022).

In recent years, renewable energy use has increased due to changing climatic conditions and energy demands (Zhang et al., 2022). Climate change has brought new challenges to horticulture since the quality of horticultural crops depends on several climatic factors (Sharma et al., 2022). For example, how to produce high-quality horticultural produce sustainably, improve crop tolerance to high (heat), chilling, or freezing temperatures, sustain crop growth under drought stress, reduce peat moss use in container substrates as peat mining releases considerable GHGs (Cleary et al., 2005), and maintain crop productivity with minimal damage from insects or pathogens (Gregory et al., 2009)—all of which require new strategies to improve food production to ensure global food security (Zhang et al., 2022). Producing horticultural crops by amending soils or container substrates with selected BC has great potential for counteracting the challenge of climate change and producing horticultural products in an environmentally friendly manner.

**Figure 1:**
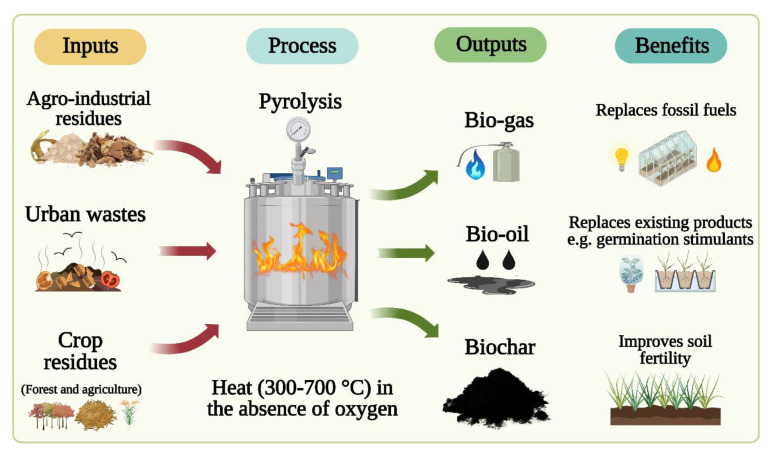
Illustration of biochar (BC) manufactured from various solid waste materials (left) and the transformation of these waste materials into useful products *via* pyrolysis (middle) and the associated horticultural benefits (right).

Biochar is a solid charcoal-like carbonaceous material produced through a thermochemical conversion process of organic biomass under the quasi-absence of oxygen at temperatures ranging from 350 to 900°C, a process referred to as pyrolysis (Lehmann and Joseph, 2009; Fan et al., 2021). This process converts the agricultural, industrial, or urban organic matter into different fractions of solid (biochar), gas (syngas), and liquid (bio-oil) products (**Figure 1**). The physical and chemical properties of BC vary substantially depending on feedstocks, temperatures, pyrolysis methods, and feedstock modifications (Yu et al., 2019). The physical properties of BC include surface area, density and porosity, pore size and volume, hydrophobicity, and water-holding capacity. In general, surface area, porosity, and pore volume increase with increased pyrolysis temperature. Biochar surface area ranges from 100 to 800 m^2^/g, and porosity ranges from 50% to 70%. Bulk density is rather low, <0.6 g/cm^3^ (Yu et al., 2019). Hydrophobicity is associated with surface function groups, and water-holding capacity depends on porosity. Chemical properties of BC include pH, cation exchange capacity, elemental composition, and surface functionality. Biochar pH and cation exchange capacity increase with increasing pyrolysis temperatures. Biochar pH varies from 5.9 to 12.3 (average 8.9). The elemental composition of BC depends on feedstock and pyrolysis temperature. The BC surface is important as various biological and chemical activities are related to surface functional groups, free radicals, surface charge, and structure-related functionality (Yu et al., 2019). Functional groups include acyl, amido, azyl, carbonyl, carboxyl, ether, ester, hydroxyl, and sulfonic (Yang et al., 2019), with critical roles in regulating pH, cation exchange capacity, nutrient and gas adsorption, contaminant degradation, and soil microbial community interactions.

**Figure 2:**
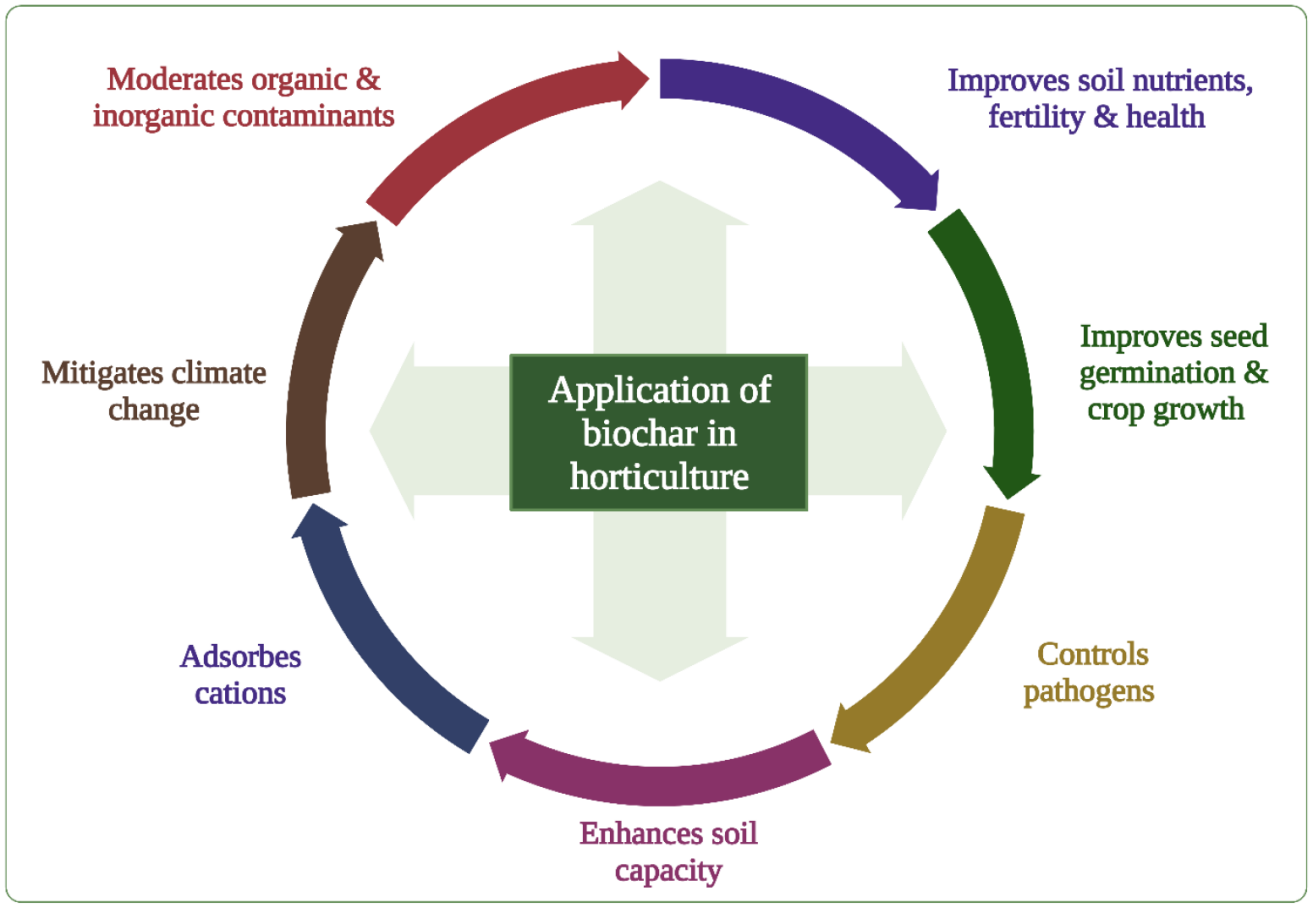
Multifunctional role of BC in horticulture sector demonstrates a positive impact of BC for ensuring food security under the threat of climate change.

Biochar has a multifunctional role, including GHG sequestration, improving soil properties, seed germination, crop yield and quality of horticulture produce, and increasing crop tolerance to abiotic and biotic stresses (Kochanek et al., 2016; Almaroai and Eissa, 2020; Zulfiqar et al., 2021a; Zulfiqar et al., 2021b; Ibrahim et al., 2022; Ji et al., 2022; Taqdees et al., 2022) (**Figure 2**). This review summarizes current progress on using BC to improve soil and substrate properties, seed germination, root growth, water and nutrient absorption, photosynthesis, crop yield, and tolerance to plant pathogens. The review also illustrates how BC can mitigate the adverse impacts of abiotic stresses on horticultural crops under climate change and discusses research gaps and the prospects of BC use for achieving high-quality maximum yields.

## Biochar application sequestrates greenhouse gases, improves soil properties, and reduces peat moss use in substrates

### Sequestration of greenhouse gases

**Figure 3:**
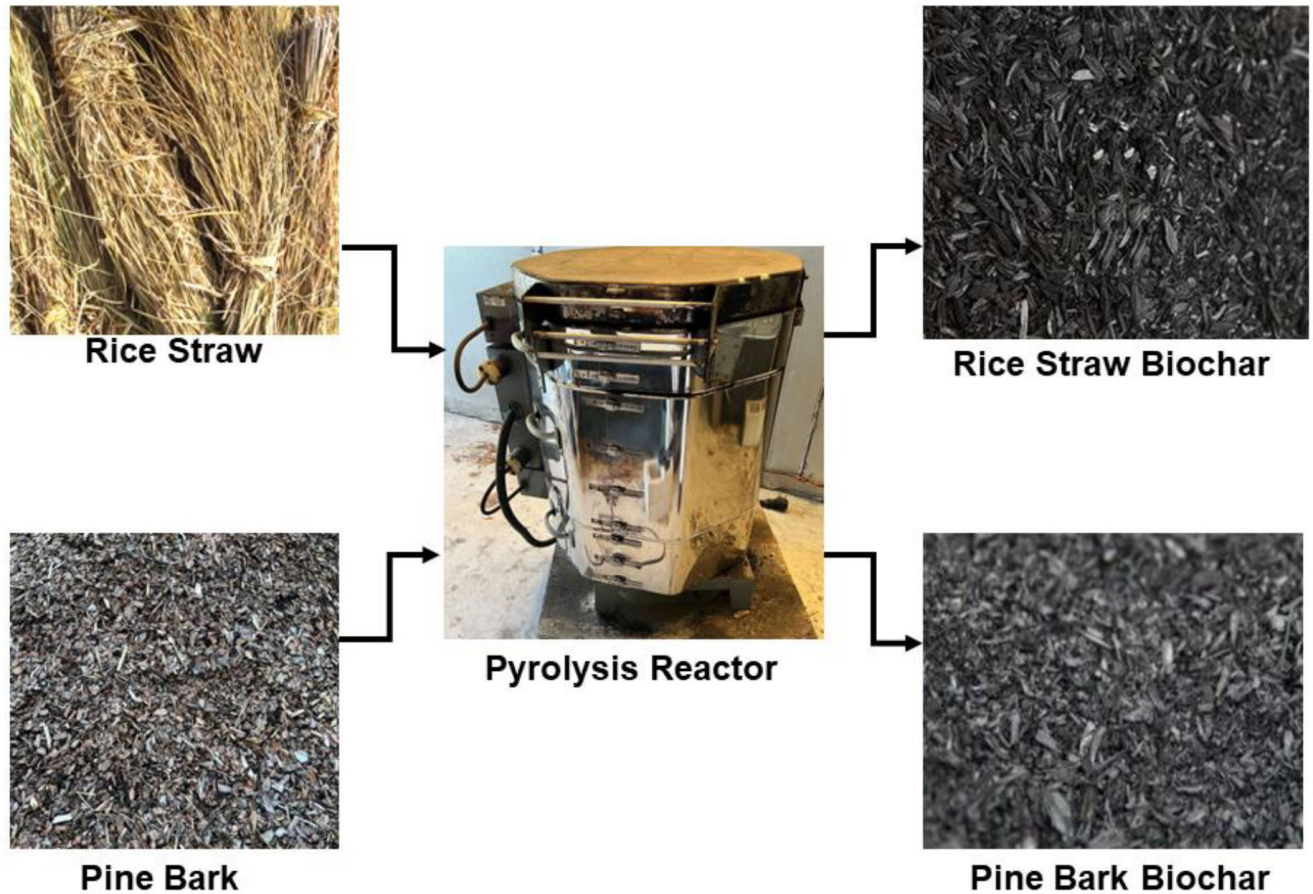
Various biochars made from agricultural waste material in a pyrolysis reactor.

Production of BC reduces CO_2_ in the atmosphere because the decay or decomposition of organic materials naturally omits CO_2_ into the atmosphere, while converting those materials into carbon-neutral products eliminates the decomposition process. **Figure 3** illustrates how BC is made from different agricultural materials. Woolf et al. (2010) showed that sustainable implementation of BC could offset up to 12% of global anthropogenic CO_2_. Composting manure is a major GHG source, but incorporating 10% (w/w) BC during composting reduced GHG emissions substantially (Yin et al., 2021). Application of BC to soil facilities for carbon capture and storage (CCS) is associated with carbon sequestration, soil, and water remediation, decreased GHG emissions, improved soil fertility, crop production, and waste management and recycling (Haider et al., 2022). Compared to other organic materials, the internal structure of BC has a remarkably high degree of stability, providing long-term soil fertility (Agegnehu et al., 2017). Mixing BC in soil at 13.5 t/ha (3% of the upper 30 cm layer) can provide carbon storage space for two centuries (Matovic, 2011). Biochar application to horticultural fields will also contribute to CCS. However, one study showed that BC application increased soil CO_2_ fluxes and reduced N_2_O fluxes with no effect on CH_4_ fluxes (He et al., 2017). The variable results could be attributed to multiple factors, including the differences in physical and chemical properties of BC and soil, variations in crop species in the tested field, climate differences, and the duration of the reported studies. The incorporation of BC into horticultural substrates mitigates CO_2_, CH_4_, and N_2_O emissions. In a microcosm incubation study, total CO_2_, CH_4_, and N_2_O emissions decreased by 50%, 21%, and 66%, respectively, in a substrate amended with biochar compared to one without BC (Lévesque et al., 2018).

### Improved soil physical, chemical, and biological properties

**Figure 4:**
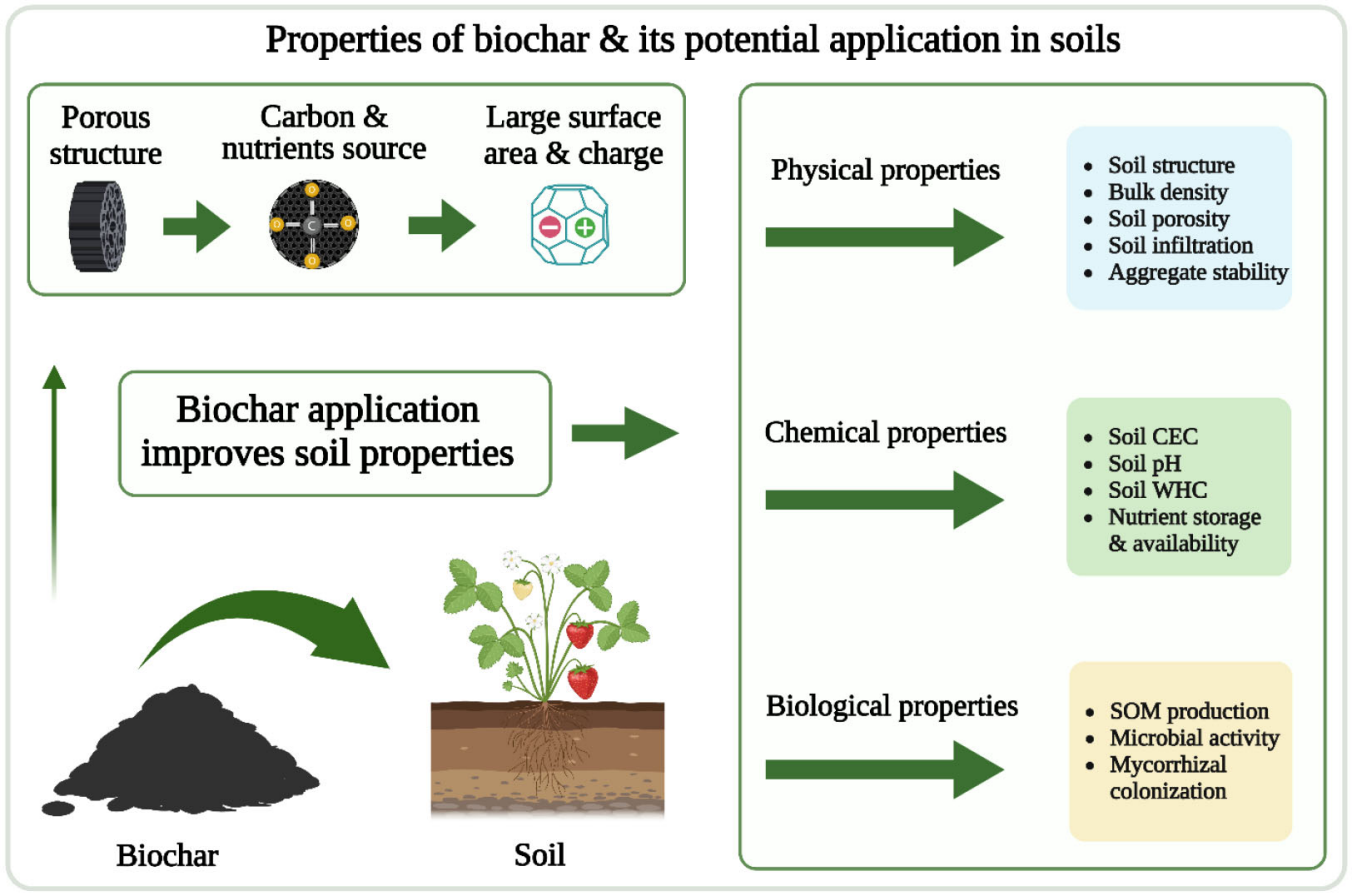
A visual demonstration of biochar impacts on soil properties.

Biochar application can improve soil physical, chemical, and biological properties (Yu et al., 2019) (**Figure 4**). A meta-analysis indicated that BC amendment decreased soil bulk density by 3%–31% (mean 7.6%) in 19 of 22 soils (Omondi et al., 2016). Fruit production usually occurs in hilly and low mountain areas where soil compaction is a problem. Peake et al. (2014) reported that BC addition reduced soil compaction by more than 10%. Soil bulk density is negatively associated with soil porosity, so a decreased bulk density increases soil porosity. Biochar application can increase soil porosity by 8.4% (Omondi et al., 2016) and improve soil structural quality and aggregation (Blanco-Canqui, 2017). In general, soil water retention improves with reduced bulk density, increased porosity, and improved structural quality.

Biochar application can increase pH in acidic soils, improve soil organic carbon, increase nutrient holding capacity due to the role of functional groups, reduce the adverse effects of heavy metal adsorption, and increase the nutrient elements supplied by the BC (Yu et al., 2019; Chang et al., 2021). A meta-analysis of 59 reports published from 2012 to 2021 showed that BC application increased soil pH, cation exchange capacity, and organic carbon by 46%, 20%, and 27%, respectively (Singh et al., 2022). Biochar application to soils also increased the relative abundance of bacteria involved in soil carbon and nitrogen cycles, soil organic carbon decomposition, and plant disease suppression and significantly increased invertase and catalase activities (Li et al., 2021). Biochar application resulted in favorable conditions for soil microorganisms, improving microbial biomass carbon (C_mic_) (Luo et al., 2014). Biochar application has been reported to relate to unique characteristics of BC, including altered root growth strategy, rhizosphere soil nutrient availability (Liu et al., 2022), and improved soil enzyme activities (Li et al., 2022a).

### Biochar as a component of container substrates

**Figure 5:**
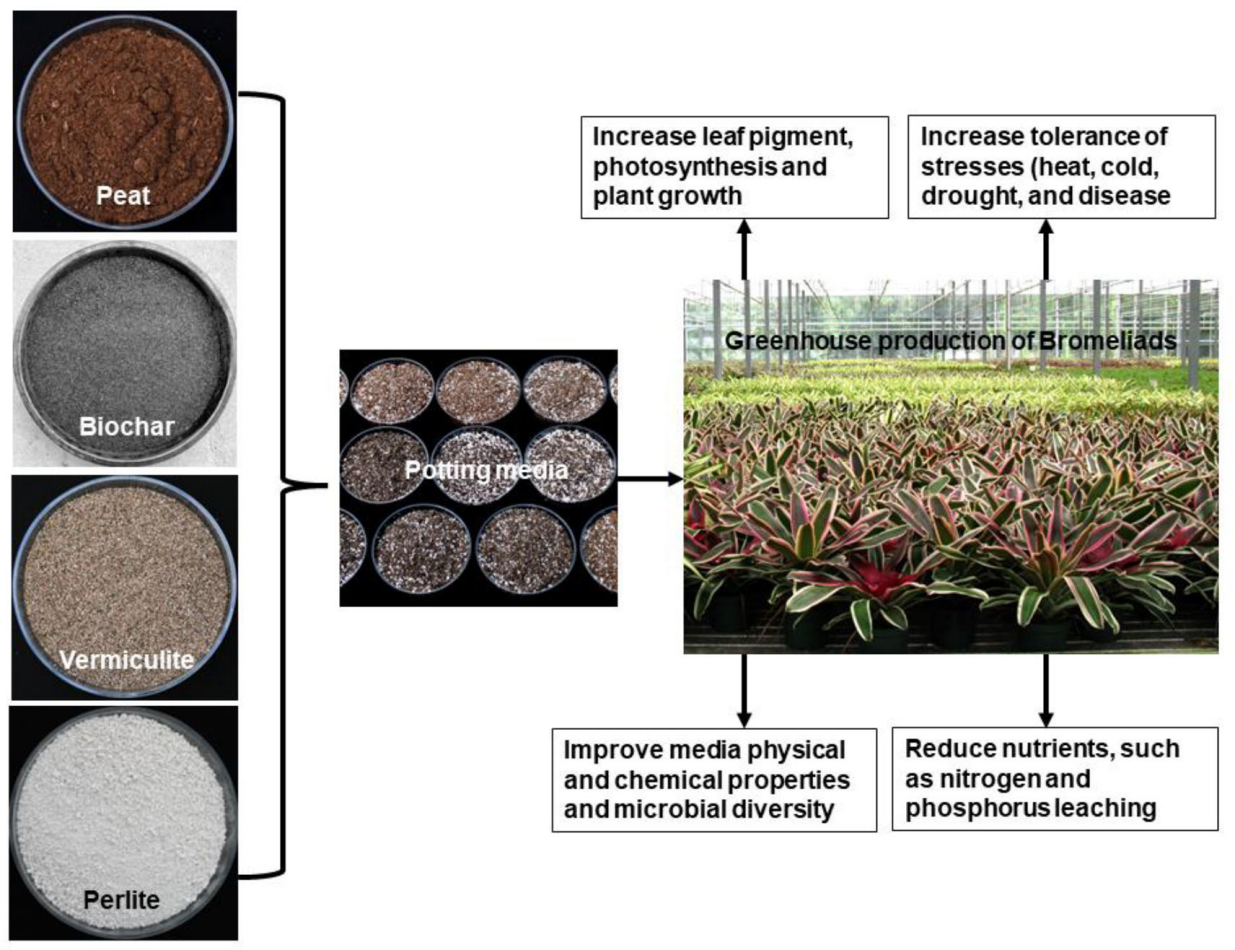
Biochar inclusion as an organic component of growing media could replace the non-renewable resource (peat), resulting in a sustainable and environmentally friendly soilless growing media.

Peat is commonly used for horticultural purposes. However, the resultant depletion of peatlands has led to an increasing environmental concern about non-renewable resource peat extraction (Zulfiqar et al., 2019a; Zulfiqar et al., 2019b). Hence, there is a continuous search for sustainable growing media substitutes, such as biochar (**Figure 5**). Ferlito et al. (2020) evaluated conifer wood BC as a growing medium component for citrus nurseries. The authors found that BC (25%) could be used in growing media comprising 50% sandy volcanic soil and 25% black peat or combined with compost (1:1) for healthy citrus production. In *Lavandula angustifolia* potted plants, Fascella et al. (2020) incorporated different BC concentrations (0, 25, 50, 75, and 100%, by volume) into peat mixtures. The treatments with 25, 50, and 75% BC improved agronomic traits and flower production in *L. angustifolia*. In lettuce (*Lactuca sativa*), Mendez et al. (2017) replaced 10% (by vol.) of peat with sewage sludge BC, improving biomass production by 184%–270% compared with 100% peat-based substrate due to enhanced microbial activities and NPK concentrations. Choi et al. (2018) reported that growing medium mixes comprising 20% pine bark and 80% BC (by vol.) boosted fresh and dry weights in chrysanthemum (*Chrysanthemum nankingense*) compared to the control with no BC. In the potted houseplant *Syngonium podophyllum*, Zulfiqar et al. (2019b) reported that BC incorporation (up to 20%) improved plant growth by improving leaf gas exchange, NPK concentrations, total soluble proteins, and soluble phenolics in leaves. Lévesque et al. (2020) reported improved growth of tomato and sweet pepper in peat-based growing media incorporating 10% or 15% (v/v) BC, with improved plant water use efficiencies, N and P uptake efficiencies, and increased fruit dry weights. In potted *Alpinia zerumbet*, Zulfiqar et al. (2021b) evaluated the impact of BC incorporation in a peat–perlite-based growing medium; growth improved with 30% BC (v/v) alone or in combination with 5% compost by regulating NPK uptake and leaf gas exchange traits. In *Rhododendron delavayi* Franch., Bu et al. (2022) evaluated the impact of two BC (v/v) applications in peat-based growing media, reporting improved plant and root growth, photosynthesis, and contents of carotenoids, polyphenols, and anthocyanins. The authors suggested using peat-based substrates containing 20% wood chip BC or 30%–40% rice husk BC for good growth.

Leaching nutrients, particularly N and P, from container substrates is a concern in ornamental plant production (Chen and Wei, 2018), as continuous leaching year-round can cause surface and/or groundwater contamination. Engineered Mg-enriched biochar can adsorb >100 mg P/g substrate (Yao et al., 2013), and Mg- and Fe-enriched LDH (layered double hydroxide) biochar can adsorb nitrate close to 25 mg/g (Xue et al., 2016). Biochar application to soil can reduce nitrate and ammonium leaching (Karhu et al., 2021). Thus, selected biochars could be used to sustain nutrients in substrates and soils and reduce leaching.

## Biochar improves seed germination of horticultural crops

Seed germination is an integral process in horticultural production, defining initial crop growth vigor and plant density to achieve optimal production and profit margins (Zulfiqar, 2021). Studies related to BC impacts on germination vary from inhibitory to stimulatory in nature depending on feedstock, pyrolysis temperature, quantity used, and crop species. Generally, positive impacts are related to low rather than high BC doses (Bai et al., 2022). Incorporating BC into soil or growing media can improve or alter porosity and water-holding capacity, enhancing seed germination *via* improved water availability. Salts and phytotoxins in BC negatively affect seed germination by inducing osmotic stress. BC triggers seed germination by releasing karakins, or germination hormones (Kochanek et al., 2016). BC incorporation also affects the physio-chemical properties of soil, promoting seed germination. For example, Jabborova et al. (2021b) reported that seed germination of sweet basil (*Ocimum basilicum*) doubled with 1% BC incorporation, while 2% and 3% BC increased germination by 28%–30%, compared with the control without BC. Not only pure BC but BC combined with various nanoparticles also promotes seed germination. Taqdees et al. (2022) reported that BC enriched with silicon nanoparticles (Si-BC) and zinc nanoparticles (Zn-BC) enhanced the seed germination of salt-stressed radish. Thus, BC can play a vital role in the seed germination of different horticultural crops under normal and stressed conditions. However, the extent of seed germination improvement depends on BC application rate, BC type, plant species, and environmental conditions.

## Biochar regulates plant water, nutrient relations and enhances root growth of horticultural crops

Plant water relations, such as water potential, osmotic potential, and turgor potential, depend on water availability and uptake and the quantity of soluble solutes. Moreover, the addition or application of organic or inorganic solutes may alter plant water relations. For example, Guo et al. (2021a) reported improved root hydraulic conductance and leaf water potential (Ψl) in water-stressed tomato grown on a sandy loam soil incorporated with miscanthus straw BC. In another study, Langeroodi et al. (2019) reported improved leaf relative water content in pumpkin in response to BC application under water deficit irrigation. Furthermore, tomato plants under deficit and partial root-zone drying irrigation supplemented with BC increased relative water content and water use efficiency (Akhtar et al., 2014). Despite the results above, further investigations are needed to confirm whether BC supplementation alters plant water relations parameters.

BC incorporation into soils and soilless growing media for horticultural crops sensitive to nutritional deficiencies can be an effective strategy to improve plant nutrition under non-stressed and stressed conditions. In drought-stressed faba bean (*Vicia faba* L.), BC application improved leaf N, P, K, and Ca^2+^ contents and decreased leaf Na^+^ content and the Na^+^/K^+^ ratio (Abd El-Mageed et al., 2021). Leaf and root mineral nutrients increased in response to BC application in cabbage (*Brassica oleracea* var. capitata) under deficit irrigation (Yildirim et al., 2021). Yang and Zhang (2022) reported that BC supply affected soil nitrogen cycling by inhibiting related bacteria, leading to enhanced N, P, K, and Mn uptake in Chinese cherry (*Prunus pseudocerasus*). In the tea plant (*Camellia sinensis* Kuntze), BC improved leaf and stem P, K, and Mg concentrations while reducing heavy metals such as Mn and Cu by altering soil pH (Yan et al., 2021). Under deficit and partial root-zone drying irrigation, BC increased the leaf C:N ratio in tomato plants but had no significant impact on N and C (Akhtar et al., 2014). The enhanced nutrient uptake by plants could be attributed to the increased nutrient supply provided by applied BC, BC-mediated changes in the rhizosphere that improved soil microbial communities, and increased bioavailability of nutrient elements.

Incorporating BC in soil and soilless growing media positively affects root growth traits. Chiomento et al. (2021) reported improved root size and biomass of strawberry (*Fragaria* × *ananassa* Duch.) treated with the combined application of BC and *Claroideoglomus etunicatum* compared to untreated plants. Likewise, in sweet basil, Jabborova et al. (2021b) reported that BC application enhanced total root length, root surface area, root volume, and root diameter compared with control plants. Biochar application increased root and shoot weights, root length and volume, and root/shoot ratio of summer savory (*Satureja hortensis* L.) under normal and salt-stressed conditions (Mehdizadeh et al., 2020). Improved root length was also reported in salt-stressed cowpea (*Vigna unguiculata* (L.) Walp.) in response to BC application (Farooq et al., 2020). Similarly, Wang et al. (2019) studied the impact of BC on apple seedlings, reporting higher root surface area (58%), root length (57%), and root volume (63%) than control plants. Moreover, apple seedling root respiration increased with increasing rates of BC application, with 745, 863, 960, and 1,239 nmol O_2_/min/g FW in the 0, 5, 20, and 80 g/kg BC treatments, respectively. The above-mentioned studies indicate that BC application affects root growth, a vital trait for promoting overall plant growth. However, how BC metabolically affects root growth remains unknown and requires further research.

## Biochar alters phytohormones and improves the photosynthesis of horticultural crops

### Altering phytohormones in horticultural crops under normal and stressed conditions

Applied BC can interact with plant growth regulators, enhancing plant growth. In a study conducted with Chinese cherry “Manaohong” (*P. pseudocerasus*), BC application increased indoleacetic acid (IAA), gibberellin A3 (GA), and zeatin levels and decreased abscisic acid (ABA) levels compared to non-treated plants. Moreover, high-throughput sequencing analysis showed the activation of IAA and inactivation of ABA signal transduction at the gene level, revealing that BC improves plant growth by regulating phytohormone signaling (Yang and Zhang, 2022). Langeroodi et al. (2019) reported that BC application decreased ABA in pumpkin seedlings under deficit irrigation relative to control plants. Akhtar et al. (2014) reported that a cotton seed shell and *Oryza sativa* husk BC mixture decreased ABA in potted tomato plants subjected to deficit and partial root-zone drying irrigation. While more research is needed, the positive interactions of BC with IAA, GA, and zeatin may indicate that phytohormone availability could increase plant growth.

### Influencing biosynthesis of photosynthetic pigments

Photosynthetic pigments affect plant photosynthetic rates and are vital determinants of healthy growth and productivity. Application of BC to horticultural crops enhances photosynthetic pigments and rates under normal and stressed conditions. For instance, Yang and Zhang (2022) reported increased chlorophyll content of the ornamental plant *Centaurea cyanus* L. in response to BC supplementation relative to control plants. Likewise, BC and humic acid treatments improved chlorophyll content in calendula (*Calendula officinalis* L.) compared to controls (Karimi et al., 2020). In fenugreek (*Trigonella foenum-graecum*), Jabborova et al. (2021a) reported that BC increased chlorophyll pigments by 30% and carotenoids by 60%. In *Berberis integerrima*, BC applications recovered the chlorophyll contents in Cd-stressed plants compared to non-treated plants. Jabborova et al. (2021c) also reported that BC increased chlorophyll pigments by 35% in ginger (*Zingiber officinale*) compared to the control. In salt-stressed cowpea (*V. unguiculata* (L.) Walp.), BC improved chlorophyll content by 6% and by 76% when combined with osmopriming (Farooq et al., 2020). In drought-stressed perennial ryegrass (*Lolium perenne* L.), rice husk BC improved chlorophyll content and drought stress tolerance. *S. podophyllum* plants grown in soilless growing media amended with BC had increased chlorophyll content compared to the non-amended control (Zulfiqar et al., 2019b). In tomatoes, Massa et al. (2019) reported decreased chlorophyll SPAD values under normal and nutritional stress conditions using BC. Similarly, Zulfiqar et al. (2021b) reported decreased chlorophyll content in *A. zerumbet* in response to BC supplementation in a soilless growing medium. SPAD values were also higher in tea plants in response to BC application than in untreated plants (Yan et al., 2021). In sweet basil, BC applications, especially at higher concentrations (2% and 3%), improved chlorophyll contents compared to low concentrations (1%) and control plants (Jabborova et al., 2021b). In sum, BC applications can improve photosynthetic pigments in different plants. However, further studies are needed to elucidate the specific mechanism involved.

### Improving leaf gas exchange characteristics

As mentioned above, BC application generally increases photosynthetic pigment contents and thus enhances leaf gas exchange characteristics. For example, Guo et al. (2021b) showed that BC application to tomato plants under deficit irrigation improved leaf gas exchange, as exemplified by the higher *P_n_*, *T_r_*, and *g_s_* than control plants. Wang et al. (2021) reported that BC significantly improved Pn, Tr, gs, and water use efficiency in field-grown peanut. In cabbage seedlings under water deficit, a low BC concentration (5%) enhanced *g*_s_, *P_n_, C_i_*, and *T_r_* more than a high BC concentration (10%). A wheat straw-based BC application upregulated leaf gas exchange traits (*g_s_*, *P_n_*, *C_i_*, and *Tr*) and carboxylation use efficiency in *A. zerumbet* grown in a sandy loam soil (Zulfiqar et al., 2021a). In tea, BC application improved the photosynthetic rate by 17% compared to untreated control plants (Yan et al., 2021). Langeroodi et al. (2019) reported that BC improved leaf gas exchange traits in pumpkin (*Cucurbita pepo*) relative to control plants. Likewise, cotton seed shell and rice husk BC increased the leaf photosynthetic rate in potted tomato plants subjected to deficit and partial root-zone drying irrigation (Akhtar et al., 2014).

## Biochar improves horticultural crop growth, yield, and produce quality

Plant growth and development rely on the health of initial starting materials, such as seeds or propagules, soil water and nutrient availability, water and nutrient absorption, and photosynthesis under the required light and temperature conditions. Biochar application to soil or growing media at an appropriate rate improves seedling growth, affects some phytohormones, and increases photosynthetic pigments and photosynthesis. As a result, studies have shown that exogenous BC application improves growth and yield in plants grown under non-stressed or stressed conditions. For example, Zulfiqar et al. (2021b) reported improved growth characteristics (plant height, tiller number, shoot diameter, and leaf number) in an important medicinal plant, *A. zerumbet*, in response to wheat straw-based BC alone and combined with compost. Jabborova et al. (2021a) found that BC application improved plant height, leaf length, leaf number, and leaf width in sweet basil relative to control plants. Wang et al. (2019) reported improved plant height, root and shoot dry weights, and root/shoot ratio in apple seedlings in response to different BC doses. Moreover, BC application to sandy soil improved the growth, dry matter, and pod yield of green pea (*Pisum sativum* L.) under low water availability (Youssef et al., 2018). In a field study, 2,544–2,625 kg/ha of BC-based fertilizer improved fertilizer efficiency and provided economic benefits in eggplant production relative to traditional fertilization practices (Zhang et al., 2022). However, the optimum BC application rates for economic profit are site- and crop-specific. For example, in a 3-year field experiment on sugar beet production, 10 t/ha BC had the maximal economical profit, increasing net profit by 6.94 billion dollars compared with no BC application (Li et al., 2022b). BC incorporation into soilless growing media is increasing due to environmental concerns associated with peat use in potted plant production. Incorporating up to 50% BC maintains the growth of containerized plants and reduces peat use (Regmi et al., 2022). Biochar not only improves plant growth and yield under normal, non-stressed conditions but also under different stresses, as discussed in the next section.

Despite improving crop growth and yield, BC also improves product quality. In tomato, cotton seed shell, and *O. sativa* husk BC increased fruit quality in terms of titratable acidity in potted tomato plants subjected to deficit and partial root-zone drying irrigation. Similarly, Agbna et al. (2017) reported increased titratable acidity, vitamin C content, and sugar/acid content ratios in tomato fruits in response to BC addition under different irrigation levels without affecting total soluble sugars. In another study, Suthar et al. (2018) reported that bamboo biochars pyrolyzed at 300 °C and amended at 3% or 450 °C and amended at 1% promoted tomato plant growth and significantly increased glucose, fructose, soluble solids, and ascorbic acid contents and sugar-to-acid ratios in fruits compared to other treatments, including the control with no biochar. Improved growth and fruit quality were related to higher concentrations of NO_3_, P, Ca, and Mg in the growing medium. In polluted soil, BC application increased the total acidity, total soluble sugars, vitamin C, and lycopene in tomato fruit compared to no biochar (Almaroai and Eissa, 2020). Application of BC increased total sugars and flavonoids by 8% in sweet basil compared to control plants (Jabborova et al., 2021b). In field experiments, BC-based fertilizer amendments increased eggplant yield and quality, including vitamin C and soluble sugar contents (Zhang et al., 2022).

## Biochar enhances the stress tolerance of horticultural crops

**Figure 6:**
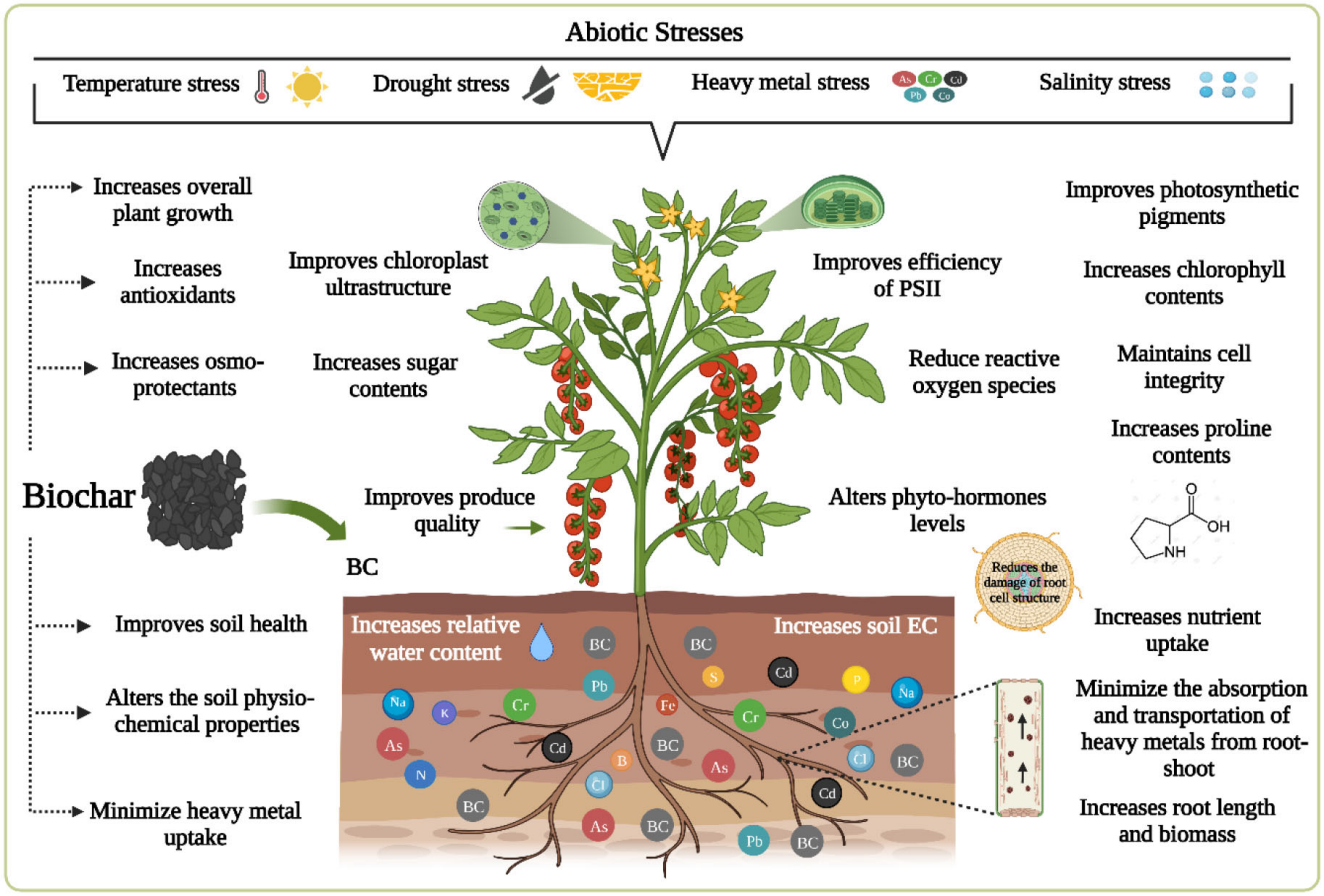
Positive impact of BC incorporation on plants under normal and stressed conditions.

Environmental conditions such as drought, salt, heavy metals, low or high temperatures, and acidic stress impair plant growth and productivity, adversely affecting overall agricultural sustainability and threatening global food security. Biochar supplementation is an efficient and cost-effective management tool for improving crop productivity in terms of increasing yield and quality. A few studies have focused on the application of BC to plants grown under stress conditions, reporting the beneficial effects of BC on regulating the myriad metabolic processes involved in growth promotion (**Table 1**). **Figure 6** is a schematic illustration of the positive effects of BC on horticultural crops under abiotic stress.

**Table 1:** The ameliorative effect of biochar on horticultural crops grown under different environmental stresses.

Name	Stress type and level; experiment type	Feedstock and concentration used	Pyrolysis temperature	Functions under stress	References
Faba beans (*Vicia faba*)	Drought stress (100, 80, and 60% evapotranspiration; field experiment	3:100 (w/w) combination of citric acid, and citrus wood biochar; 0, 5, or 10 ton ha^−1^	Information not mentioned	Improved plant growth and physiological responses Enhanced contents of N, P, K, and Ca.	Abd El-Mageed et al., 2021
Pumpkin (*Cucurbita pepo* L.).	Drought stress (60%, 75%, and 90%); field experiment	Maize-straw biochar 0, 5, 10 and 20 ton biochar ha^−1^	350°C For 7 days	Increased chlorophyll Enhanced uptake of nutrients Reduced reactive oxygen species	Langeroodi et al. (2019)
Tomato (*Solanum lycopersicum*)	Deficit irrigation and partial root drying	Mixture of rice husk and shell of cotton seed biochar; 0% and 5% by weight	400°C	Improved photosynthesis Enhanced relative water content and water use efficiency Improved fruit yield and quality Decreased membrane stability index Decreased ABA content.	Akhtar et al. (2014)
Cabbage (*Brassica oleracea*)	Drought stress (100% and 50% irrigation); pot experiment	Commercial biochar comprising 60% sewage sludge and 40% domestic wastes; 0%, 5%, or 10%	1st stage reactor was kept at 150°C under 5–8 bar Pressure; 2nd stage reactor (hydrolysis) (250°C) and pressure (50 bar); 3rd stage reactor (cracker): 550°C),	Recovered plant growth Improved leaf gas exchange traits Reduced MDA, H_2_O_2_, proline, and sucrose content Enhanced mineral nutrient content of leaf and root	Yildirim et al. (2021)
Summer savory (*Satureja hortensis* L.)	Salt stress (0, 3, 5, and 7 dS m^−1^); pot experiment	Woody branches of *Morus alba* L.; 0, 1% and 2% of total pot	530°C temperature for 14 h	Improved fresh and dry biomass Improved root morphological traits Enhanced leaf SPAD value Increased proline content Decreased membrane injury Reduced Na^+^ and Cl^-^ contents	Mehdizadeh et al. (2020)
*Potato* (*Solanum tuberosum*)	Salt stress; field experiment	Rice husk; 825 kg/ha	Pyrolyzed in an oven at 350°C for 24 h	Improved plant growth Enhanced leaf gas exchange traits Enhanced gibberellic acid and decreased abscisic acid	Mahmoud et al. (2022)
Cow pea (*Vigna unguiculata* (L.) Walp.)	Salt stress; 10 dS m^−1^; pot experiment	Mango wood biochar; plastic pots (150 mm × 90 mm) filled with peat moss having biochar (25 g per pot)	500°C for 30 min	Improved shoot length, root length, plant biomass and leaf area Improved chlorophyll contents, amylase activity, total soluble sugars Decreased Na+ uptake, MDA, and	Farooq et al. (2020)
(*Brassica oleracea* var. capitata)	Salt stress; 0 and 150 mM NaCl; pot experiment	60% sewage sludge and 40% domestic wastes; 0%, 2.5%, and 5% by soil weight	Depolymerization at 150 °C under 5–8 bar pressure; Hydrolysis at high temperature (250°C) and pressure (50 bar), cracker stage at 550°C	Improved plant growth and biomass Enhanced chlorophyll Decreased MDA, H_2_O_2_, sucrose and proline Enhanced leaf nutrient elements Decreased Na and Cl content in plant	Ekinci et al. (2022)
Tomato (*Solanum lycopersicum*)	Metal contaminated soil; Field experiment	Maize stalks biochar; 0, 5, or 10 ton ha^−1^	Muffle furnace, set at a heating rate of 15°C min^–1^ for 2.5 h at a peak temperature of 450°C.	Improved chlorophyll contents Improved yield and fruit quality Decreased uptake of heavy metals in both plant parts and fruits	Almaroai and Eissa (2020)
Lettuce (*Lactuca sativa* L.)	Copper contaminated soil; pot experiment	Orchard pruning feedstock based commercial biochar; 1% biochar (w/w)	550°C, slow-pyrolysis	Restored antioxidant activity and flavonoids Increased total phenols, phenolic acids and anthocyanins	Quartacci et al. (2017)

### Enhancing drought tolerance

Incorporating BC into growing media or soil can improve crop tolerance to drought stress. Wood pellets BC added to a growing medium (30% v/v) improved tomato seedling tolerance to drought (Mulcahy et al., 2013). Agbna et al. (2017) reported that water-stressed tomato plots amended with 25 t/ha of wheat straw BC had similar fruit yields as unstressed control plots, accompanied by increased plant height, leaf numbers, and fresh and dry plant weights. Egamberdieva et al. (2017) evaluated BC-based *Bradyrhizobium* inoculum on the growth of drought-stressed lupin (*Lupinus angustifolius* L.), noting improved drought tolerance associated with revived growth, increased nutrient uptake, and improved symbiotic performance. Li et al. (2018) reported that 30 t/ha BC and deficit irrigation saved water without affecting total tomato productivity in a field experiment. In the same experiment, increasing the maize straw BC application rate from 10 to 40 t/ha increased plant dry biomass and fruit yield. However, higher BC application rates had no positive effect or decreased plant growth and yield. Moreover, a cost–benefit analysis showed that BC applications of 10, 20, and 40 t/ha positively affected net profit. In contrast, 60 t/ha negatively affected net profit compared with the unamended control. A quadratic function fitting net profit and BC application rate showed an optimal application rate of 27 t/ha (Li et al., 2018).

In a two-year study on pumpkin (*C. pepo*) under reduced irrigation, Langeroodi et al. (2019) reported that BC-amended plants had decreased ROS, an elevated antioxidant defense system, and improved photosynthesis and overall growth relative to unamended plants. Egamberdieva et al. (2020b) evaluated the impact of maize BC on drought-stressed broad beans (*V. faba* L.), reporting improved growth, yield, and nutrient uptake relative to unamended plants. Abd El-Mageed et al. (2021) studied the impact of acidified BC on drought-stressed *V. faba* under field conditions in a two-year experiment, reporting that BC addition improved plant growth relative to unamended soil. Biochar application to drought-stressed cabbage plants ameliorated the adverse effects of drought stress by increasing leaf water relative content and photosynthetic activities, with a 5% BC application improving shoot and root fresh and dry weights compared to unamended control plants (Yildirim et al., 2021). Abd El-Mageed et al. (2021) reported a 39% increase in seed yield of drought-stressed *V. faba* in BC-amended soil under field conditions compared with unamended soil. In a greenhouse experiment, Akhtar et al. (2014) reported that 5% (w/w) cotton seed shell and *O. sativa* husk BC increased the fruit yield of potted tomato plants subjected to 30% deficit and partial root-zone drying irrigation by 6% and 13%, respectively, compared to fully irrigated control plants. Improved drought tolerance with BC application could be attributed to increased porosity and pore volume in BC-amended soil, increasing the capacity to hold available water under drought stress. Further research is needed to address the effect of different soil porosities and pore volumes with BC application on crop growth under drought stress.

### Enhancing salt tolerance

An estimated 831 million ha of arable land globally is affected by salinity and is expected to increase due to ongoing irrigated farmland growth and climate change (Wu et al., 2022). Growing crops on saline soils in locations where water supply is frequently limited is necessary to fulfill the food demands of an expanding population (Zulfiqar, 2021). Biochar incorporation into soil can help ameliorate the adverse impacts of salt stress on plants (Egamberdieva et al., 2022). For example, Mehdizadeh et al. (2020) reported that BC application improved growth and leaf mineral nutrients and decreased leaf Na^+^ and Cl^–^ contents in salt-stressed summer savory. In cowpea (*V. unguiculata* (L.) Walp.), BC application improved salt stress tolerance by elevating chlorophyll content, amylase activity, and total soluble sugars and decreasing Na^+^ and MDA contents and total antioxidant activity (Farooq et al., 2020). In salt-stressed spinach, combined *Trichoderma* and BC ameliorated the adverse effect of salt stress by decreasing ROS, altering phytohormones, and improving the physiological condition of plants (Sofy et al., 2021). Ghassemi-Golezani and Farhangi-Abriz (2021) reported enhanced salt tolerance in safflower (*Carthamus tinctorius* L.) in response to BC-based metal oxide nanocomposites of manganese (Mn) and magnesium (Mg), with improved root growth, root density, plant height, plant biomass, leaf area, chlorophyll content, branch and flower numbers, potassium, Mg, and Mn contents, duration of the reproductive stage, and seed and oil yields and decreased Na^+^ content. BC can also improve plant’s ability to cope with salt-induced oxidative stress. For example, Song et al. (2022) recently reported that organic amendment incorporation, including BC, to salt-affected soil reduced oxidative stress and improved morpho-physiological conditions by promoting Na^+^ exclusion and K^+^ accumulation in hybrid *Pennisetum*, relieving stomatal restriction, increasing leaf pigment levels, electron transport efficiency, net photosynthesis, and root activity, and reducing oxidative damage.

In another study, Ghassemi-Golezani and Abdoli (2022) evaluated the impact of BC-based rhizobacteria for mitigating salt stress in rapeseed (*Brassica napus* L.), reporting improved nutrient contents, non-enzymatic antioxidants, main and lateral root lengths and weights, main/lateral root length ratio, specific root length, root diameter, shoot length and weight, leaf area, chlorophyll content, and seed and oil yields, and reduced sodium contents, ROS generation, lipid peroxidation, and enzymatic antioxidant activities in plant tissues. Ghassemi-Golezani and Rahimzadeh (2022) tested BC-based nutritional nanocomposites for improving salt stress tolerance in dill (*Anethum graveolens*), reporting decreased sodium contents that reduced the levels of osmolytes, antioxidant enzyme activities, ROS, lipid peroxidation, NADP reduction, ABA, jasmonic acid, and salicylic acid in leaves. Moreover, BC addition, particularly BC-based nanocomposites, to salt-affected soil enhanced plant organ biomass, photosynthetic pigments, leaf K, Fe, and Zn contents, leaf water content, hill reaction, ATPase activities, oxygen evolution rate, endogenous indole-3-acetic acid, and essential oil yield (Ghassemi-Golezani and Rahimzadeh, 2022). Ekinci et al. (2022) also observed BC-induced salt stress tolerance in cabbage seedlings (*B. oleracea* var. capitata), decreasing malondialdehyde (MDA), hydrogen peroxide (H_2_O_2_), proline, and sucrose contents while increasing leaf and root nutrient contents of cabbage seedlings (except Na and Cl) and regulating ABA contents. BC incorporation also causes osmotic adjustments in plants under salt stress. For instance, BC application ameliorated salt stress in borage (*Borago officinalis* L), a moderately salt-tolerant medicinal plant, by improving plant water status and osmotic adjustment capacity linked to increased photosynthetic pigments, antioxidant activation, osmolyte accumulation, and increased K^+^ content and K^+^/Na^+^ ratio (Farouk and AL-Huqail, 2022). Biochar-ameliorated salt stress tolerance could be due to: (1) BC increasing the available water holding capacity of soil and improving soil chemical properties by limiting Na availability to plant roots; (2) BC generally contains high K; and (3) both factors leading to increased K uptake and decreased Na uptake, ameliorating the adverse effects of Na. However, molecular research on BC-mediated salt tolerance is needed to provide more detailed information on salt stress tolerance driven by BC amendment.

### Enhancing tolerance to heavy metals

Heavy metal pollution in soil has become more challenging with increasing urbanization and rapid improvements in agricultural and industrial technologies, posing major threats to human health and the environment. Biochar has gained popularity as a potential substance for successfully immobilizing heavy metals in polluted soil and, thus, reducing their uptake by plants. Controlling metal absorption in horticultural crops is critical for human consumption and maintaining the productivity and quality of horticultural produce. In this context, many studies have found that BC helps reduce heavy metal stress in horticulture crops. Quartacci et al. (2017) reported that BC addition to contaminated soil restored flavonoids and antioxidant activity and increased phenolic acids, total phenols, and anthocyanins compared to the control. Almaroai and Eissa (2020) reported that BC decreased the transfer of heavy metals (Pb and Cd) from non-edible parts to edible parts of tomato (*Solanum lycopersicum*) grown in metal-polluted soil, increasing tomato yield by 20%–30%. Ibrahim et al. (2022) tested three BC types (*Casuarina*, mango, and Salix as feedstocks) for their impact on summer squash (*C. pepo* L.) grown in contaminated soil. *Casuarina*-based BC applied at low concentration (2%) had the highest growth rate, while the high concentration (4%) decreased root and shoot uptake of Cd, Co, Cr, Cu, Ni, Pb, and Zn by regulating soil pH, organic matter, and electrical conductivity. Moreover, the 4% *Casuarina*-based BC had the highest reduction in the bioconcentration and translocation factors of these heavy metals. Thus, BC amendment in contaminated soils can improve yield and the quality of produce by reducing the uptake of heavy metals such as Cd, Cu, and Zn (Tahervand et al., 2022), presenting a novel phytoremediation strategy for contaminated soils by phytostabilizing toxic metals *in situ* while reducing their bioavailability to crops and leaching into groundwater.

### Enhancing tolerance to acidic soils

Acidic soils are known for their lower crop productivity than neutral or alkaline soils, which is attributed to poor fertility, mineral toxicity (e.g., Al, Mn, and Fe), and other nutrient deficiencies. Some horticultural fruit crops and landscape plants often grow in acidic soils, significantly affecting their growth. Biochar can elevate soil pH due to its alkalinity. A BC amendment can also increase plant tolerance to soil acidity. For example, Yan et al. (2021) reported that BC application improved the growth of tea plants by enhancing leaf area, photosynthesis, and mineral nutrients in acidic soil. Yin et al. (2022) compared the effects of BC and hydrochar made from cow manure and reed straw on the growth of lettuce under acid soil conditions, reporting that cow manure-based BC amendments improved lettuce growth by increasing soil pH, P and available K contents, soil bacterial activity, and the abundance of beneficial bacteria compared to reed straw and hydrochar.

## Biochar improves plant disease resistance

Biochar application can improve plant tolerance and resistance to plant pathogens. BC contains butyric acid, ethylene glycol, 2-phenoxyethanol, benzoic acid, quinines, o-cresol, hydroxyl-propionic acid, and propylene glycol that impact soil microbiota growth. Soil amendments with low BC concentrations can suppress susceptible disease-causing species through the compounds mentioned above, which ultimately improve plant resistance and crop productivity (Graber et al., 2010; Frenkel et al., 2017). Soil BC amendment can lead to disease resistance and vulnerability in horticultural crops due to changes in plant metabolic pathways (Ji et al., 2022). BC-induced disease suppression or resistance is highly dose-dependent and BC-specific. As a strong absorbent of organic compounds from soil, BC alleviated root rot disease in Chinese ginseng (*Panax ginseng*) (Liu et al., 2022). In another study, BC addition to soil decreased *Botrytis cinerea-*induced fungal diseases of *Capsicum annuum* and *S. lycopersicum* (Mehari et al., 2015). In tomatoes, Rasool et al. (2021) noted that BC induced the expression of defense-associated genes, including jasmonic acid related *PI2*, *TomloxD*, salicylic acid-related *PR1a*, *PR2*, and ethylene-responsive *Pti4*, phenolics, CAT, and POX, to ameliorate infection-induced damage from *Alternaria solani* in tomatoes. Liu et al. (2022) reported that a BC amendment increased apple seedling growth through the absorption of apple replant disease-causing abiotic factor (phloridzin) from soil. Exogenously applying BC to *F. ananassa* improved resistance against *B. cinerea*, *Podosphaera apahanis*, and *Colletotrichum acutatum* (Meller Harel et al., 2012). Furthermore, in *Asparagus officinalis*, BC supplementation to soil decreased *Fusarium* root rot infection by 50% more than unamended soil (Głuszek et al., 2017).

Egamberdieva et al. (2020a) reported that the combined effect of BC and endophytic bacteria improved growth and controlled root rot disease incidence in Fusarium-infested narrow-leafed lupin (*L. angustifolius* L.). Molecular analysis revealed that BC application to tomato plants provided resistance against root rot and Fusarium crown by exerting a priming effect on gene expression. The study showed that BC application upregulated the pathways and genes associated with plant defense and growth, such as auxins, brassinosteroids, cytokinins, jasmonic acid, and the synthesis of cell walls, flavonoids, and phenylpropanoids (Jaiswal et al., 2020). In another study, Jaiswal et al. (2019) reported pre-emergence damping-off disease control with BC application in nursery plants. Improved disease resistance is often associated with improved soil bacterial communities (Wang et al., 2019). Application of peanut shell and wheat straw biochars to soil significantly reduced the disease index of bacterial wilt caused by *Ralstonia solanacearum* by 28.6% and 65.7%, respectively (Lu et al., 2016). Biochar can offset fungal and bacterial diseases and thwart viral diseases. For example, Luigi et al. (2022), using the RT-qPCR technique and ^−ΔΔ^Ct analysis, noted that BC addition to soil reduced spotted wilt virus and potato spindle tuber viroid (PSTVd) infection rates and virus replication in tomato.

## Conclusions and prospects

Horticultural intensification is urgently needed to feed the increasing global human population. In the post-pandemic-induced financial crisis, increased horticultural production is also needed to keep the industry profitable under the challenges of climate change and poor sustainability. Biochar is an organic material with huge potential for use as a sustainable material to meet such urgent targets in horticulture. This review discusses recent studies on the possible positive outcomes of BC in horticulture, especially those related to plant physio-biochemical mechanisms. However, studies are needed to evaluate different feedstocks at different concentrations and functional group modifications to meet specific needs for BC application. Moreover, molecular studies are needed to better understand BC-induced positive impacts on different plants. Most studies have been performed in pots; hence, future studies should be field-oriented and across more than one growing season to better evaluate BC-induced outcomes. Nano-forms of BC should be tested in horticulture. Using raw BC in soils or growing media is financially unrealistic due to the high initial costs; hence, appropriate technology needs to be developed for economic BC production to meet the long-term horticultural industry’s needs. Moreover, the increase in industrialization has exacerbated the heavy metal issue, and BC strategies should be designed to minimize the uptake of hazardous materials in fruits and vegetables. Moreover, in most developing countries, vegetable production is often close to major towns to meet local market needs and reduce transportation costs. However, it frequently relies on town sewage water, a source of heavy metals and pollutants. Hence, testing BC with sewage water for the safe production of horticultural produce would be a breakthrough technology. In addition, BC in a processed form, such as BC-based slow-release fertilizers or peat alternative growing media, should be commercialized for sustainable horticulture. Studies should also be carried out combining BC application with microbial and non-microbial bio-stimulants to open new opportunities for enhancing horticultural production. In summary, the horticulture sector can increase productivity with profitability and sustainability using BC-amendment technologies.

## Author contributions

FZ conceived the idea, prepared the first draft, and revised the manuscript. All authors listed have made a substantial, direct, and intellectual contribution to the work and approved it for publication.

## Conflict of interest

The authors declare that the research was conducted in the absence of any commercial or financial relationships that could be construed as a potential conflict of interest.

## Publisher’s note

All claims expressed in this article are solely those of the authors and do not necessarily represent those of their affiliated organizations, or those of the publisher, the editors and the reviewers. Any product that may be evaluated in this article, or claim that may be made by its manufacturer, is not guaranteed or endorsed by the publisher.
